# RimNet: A deep 3D multimodal MRI architecture for paramagnetic rim lesion assessment in multiple sclerosis

**DOI:** 10.1016/j.nicl.2020.102412

**Published:** 2020-09-04

**Authors:** Germán Barquero, Francesco La Rosa, Hamza Kebiri, Po-Jui Lu, Reza Rahmanzadeh, Matthias Weigel, Mário João Fartaria, Tobias Kober, Marie Théaudin, Renaud Du Pasquier, Pascal Sati, Daniel S. Reich, Martina Absinta, Cristina Granziera, Pietro Maggi, Meritxell Bach Cuadra

**Affiliations:** a Signal Processing Laboratory (LTS5), Ecole Polytechnique Fédérale de Lausanne, Switzerland; b Medical Image Analysis Laboratory (MIAL), Center for Biomedical Imaging (CIBM), University of Lausanne, Switzerland; c Department of Radiology, Lausanne University Hospital and University of Lausanne, Switzerland; d Neurologic Clinic and Policlinic, Departments of Medicine, Clinical Research and Biomedical Engineering, University Hospital Basel and University of Basel, Basel, Switzerland; e Translational Imaging in Neurology (ThINK) Basel, Department of Medicine and Biomedical Engineering, University Hospital Basel and University of Basel, Basel, Switzerland; f Division of Radiological Physics, Department of Radiology, University Hospital Basel, Basel, Switzerland; g Advanced Clinical Imaging Technology, Siemens Healthcare AG, Lausanne, Switzerland; h Department of Neurology, Lausanne University Hospital and University of Lausanne, Lausanne, Switzerland; i Translational Neuroradiology Section, National Institute of Neurological Disorders and Stroke, National Institutes of Health, Bethesda, MD, USA; j Department of Neurology, Cedars-Sinai Medical Center, Los Angeles, CA, USA; k Department of Neurology, Johns Hopkins University, Baltimore, MD, USA; l Department of Neurology, Cliniques Universitaires Saint-Luc, Université Catholique de Louvain, Brussels, Belgium

**Keywords:** Multiple sclerosis, Paramagnetic rim lesions, Susceptibility-based MRI, Deep learning, Supervised classification, Multimodal network

## Abstract

**Objectives:**

In multiple sclerosis (MS), the presence of a paramagnetic rim at the edge of non-gadolinium-enhancing lesions indicates perilesional chronic inflammation. Patients featuring a higher paramagnetic rim lesion burden tend to have more aggressive disease. The objective of this study was to develop and evaluate a convolutional neural network (CNN) architecture (RimNet) for automated detection of paramagnetic rim lesions in MS employing multiple magnetic resonance (MR) imaging contrasts.

**Materials and methods:**

Imaging data were acquired at 3 Tesla on three different scanners from two different centers, totaling 124 MS patients, and studied retrospectively. Paramagnetic rim lesion detection was independently assessed by two expert raters on T2*-phase images, yielding 462 rim-positive (rim+) and 4857 rim-negative (rim-) lesions. RimNet was designed using 3D patches centered on candidate lesions in 3D-EPI phase and 3D FLAIR as input to two network branches. The interconnection of branches at both the first network blocks and the last fully connected layers favors the extraction of low and high-level multimodal features, respectively. RimNet’s performance was quantitatively evaluated against experts’ evaluation from both lesion-wise and patient-wise perspectives. For the latter, patients were categorized based on a clinically relevant threshold of 4 rim+ lesions per patient. The individual prediction capabilities of the images were also explored and compared (DeLong test) by testing a CNN trained with one image as input (unimodal).

**Results:**

The unimodal exploration showed the superior performance of 3D-EPI phase and 3D-EPI magnitude images in the rim+/- classification task (AUC = 0.913 and 0.901), compared to the 3D FLAIR (AUC = 0.855, Ps < 0.0001). The proposed multimodal RimNet prototype clearly outperformed the best unimodal approach (AUC = 0.943, P < 0.0001). The sensitivity and specificity achieved by RimNet (70.6% and 94.9%, respectively) are comparable to those of experts at the lesion level. In the patient-wise analysis, RimNet performed with an accuracy of 89.5% and a Dice coefficient (or F1 score) of 83.5%.

**Conclusions:**

The proposed prototype showed promising performance, supporting the usage of RimNet for speeding up and standardizing the paramagnetic rim lesions analysis in MS.

## Highlights

• RimNet, an automated method to detect paramagnetic rim in Multiple Sclerosis lesions.
• Different rim detection ability of 3D FLAIR and 3D EPI (T2* & Phase) MRI.
• RimNet performance is close to experts’ at lesion and patient-wise levels.
• Automated rim analysis is feasible with one single 3D EPI MR acquisition.
• Excellent RimNet performance is maintained in inter-hospital evaluation.

## Graphical abstract

**Figure:**
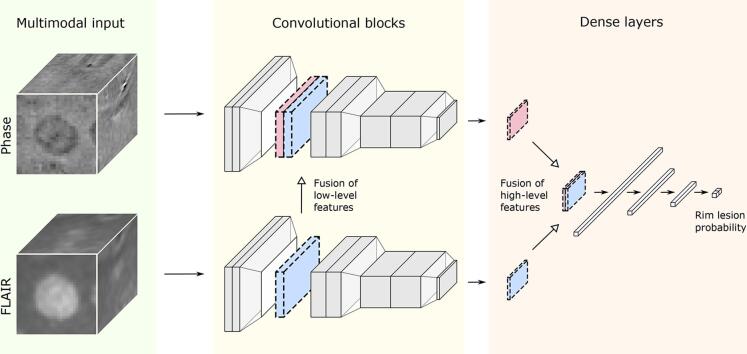

## 1 Introduction

**Fig. 1:**
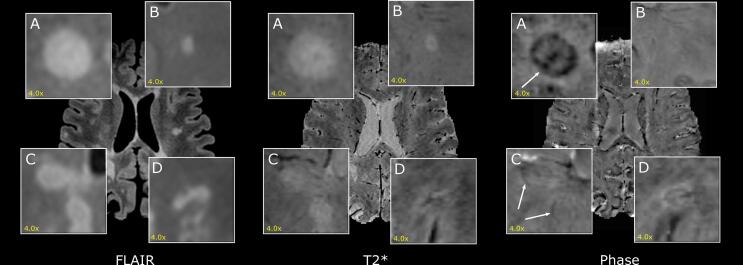
Example of MS lesions on 3D FLAIR (left), 3D-EPI magnitude (center) and phase (right) images. A) and B) are clear examples of lesions with presence (rim+) and absence (rim-) of a paramagnetic rim, respectively. In C) there are two more subtle rim+ lesions. D) is an example of a rim- lesion that has a rim+ like intensity artefact.

Multiple sclerosis (MS) is an immune-mediated disorder characterized by focal inflammatory and demyelinating lesions in the brain and spinal cord. After acute inflammatory demyelination subsides, compartmentalized/smoldering inflammation persists at the edge of some chronic MS lesions, termed “chronic active lesions.” These lesions, which are pathologically characterized by perilesional accumulation of iron-laden microglia/macrophages (Absinta et al., 2016, Dal-Bianco et al., 2017, Kaunzner et al., 2019), can be depicted with in vivo susceptibility-based MRI as non-gadolinium enhancing lesions with a paramagnetic rim (see Fig. 1A) (Hammond et al., 2008, Pitt et al., 2010, Bagnato et al., 2011, Hagemeier et al., 2012, Yao et al., 2012, Walsh et al., 2013, Wisnieff et al., 2015, Harrison et al., 2016, Dal-Bianco et al., 2017, Absinta et al., 2018, Kaunzner et al., 2019). From a clinical perspective, accrual of MS patient’s disability despite available disease modifying therapies is associated with a higher paramagnetic rim lesion burden (Harrison et al., 2016; Absinta et al., 2019). So far, routine imaging protocols can only detect MS acutely inflamed gadolinium-enhancing lesions, and no tools are available to depict chronically inflamed lesions. Moreover, in progressive MS patients, conventional radiological markers of disease activity (such as new T2 lesions or gadolinium-enhancing lesions) are rarely detectable. For all these reasons, the paramagnetic rim MRI biomarker might be used for patient stratification and potentially serve as an outcome measure in MRI based clinical trials in the future ( Absinta et al., 2019).

Imaging protocols for the paramagnetic rim analysis usually include a T2-weighted sequence (such as 3D FLAIR (Chagla et al., 2008)) for lesion detection and a susceptibility-based sequence (such as T2*-weighted, T2*-*w*, magnitude and phase, susceptibility-weighted imaging, or quantitative susceptibility mapping) to classify lesions based on whether a paramagnetic rim is visible or not (Hagemeier et al., 2012, Yao et al., 2012, Absinta et al., 2018, Kaunzner et al., 2019). Up to the present time, the presence/absence of perilesional paramagnetic rims has been determined through visual inspection by experts.

**Fig. 2:**
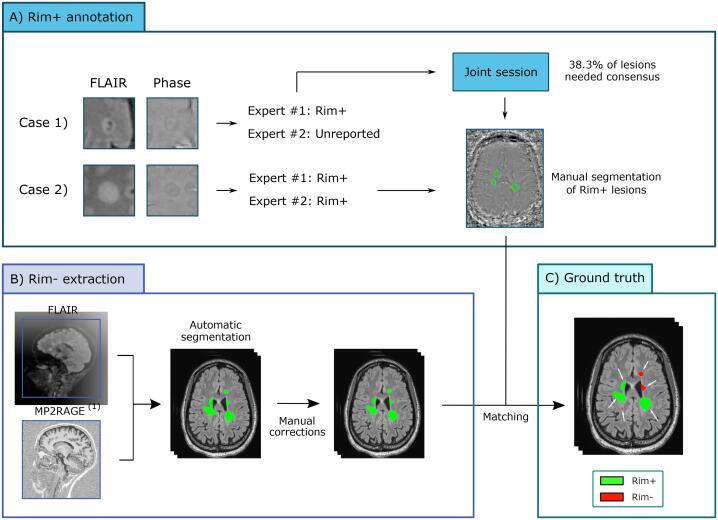
Description of the protocol used to label and generate our dataset. A) For each patient, two experts visually inspected the 3D-EPI phase and 3D FLAIR images and only reported paramagnetic rim lesions (rim+ lesions). The rim+ lesions detected by one expert and undetected or considered rim- by the other (unreported) went through a joint session where experts provided a final decision. B) Lesion candidates were extracted from the automatic segmentation (corresponding to the connected components by considering a 6-connected-voxels neighborhood) and matched with the rim+ annotations. In order to guarantee that one lesion candidate matched only one rim+ lesion annotation, a technician manually separated the rim+ lesions inside confluent ones. (1) MP2RAGE for Basel patients and MPRAGE for Lausanne patients.

A robust and accurate method to automatically detect paramagnetic rim lesions would represent a valuable decision support tool for radiologists and an opportunity to facilitate integration of this promising MRI biomarker into the MS clinical reading workflow. Moreover, given the interobserver variability observed for this particular task (Absinta et al., 2018), the integration of such method as a potential CNN second rater, would help to yield more reliable paramagnetic rim lesions assessment. To our knowledge, such an approach has not yet been explored. From a computer vision perspective, the classification of lesions based on the presence/absence of a paramagnetic rim faces three major challenges: 1) the intensity features of the rim are not necessarily discernible from the internal lesion parenchyma and/or surrounding white matter (WM) tissue (Fig. 1C); 2) some rim-like intensity artefacts may appear (Fig. 1D); and 3) the scarcity of paramagnetic rim lesions for training due to their relatively lower frequency in MS patients (imbalanced dataset with a large majority of non-paramagnetic rim lesions).

In this work, we propose the first automated method based on supervised classification to distinguish lesions featuring a paramagnetic rim (hereafter referred as rim+ or rim-). Our method is based on a 3D patch-based convolutional neural network (CNN) architecture (RimNet), which exploits different MR imaging contrasts combined at the first and last layers of the network. We have performed a multi-center and multi-scanner comparison of our CNN results and evaluated the performance (at both the lesion and patient level) in comparison to the manual annotation of two experts on a cohort of 124 MS patients.

## 2 Materials and methods

### 2.1 Participants and MRI acquisition

We retrospectively analyzed MRI scans from MS patients diagnosed according to the revised 2010 McDonald MS criteria (Polman et al., 2011) who were recruited between December 2017 and September 2019 in two academic research hospitals, the *Centre Hospitalier Universitaire Vaudois* (Lausanne, Switzerland) and the *Universitätsspital Basel* (Basel, Switzerland). Of the 141 eligible MS patients, 124 were included (11 patients were excluded because of motion artefacts and 6 because of coexisting brain pathologies): 55 patients (33 female, 25–76 years old) in Lausanne and 69 patients (44 female, 22–73 years old) in Basel. Patients' demographic and clinical characteristics are summarized in Table 1. The study received approval by the local ethics committee, and all patients gave written informed consent for the retrospective use of their data.

**Table 1:** Number of patients per hospital, mean and standard deviation of patients’ age, median and interquartile range of patients’ EDSS (Kurtzke 1983) and number of patients per MS type. Abbreviations: EDSS, expanded disability status scale; RRMS, relapsing-remitting MS; PPMS, primary-progressive MS; SPMS, secondary-progressive MS.

Hospital	Scanner	#Patients	Age (years)	EDSS	RRMS	PPMS	SPMS
Lausanne	Skyra/Prisma	55	47.8 ± 11.0	2.0 (1.5–5.0)	36	9	10
	Skyra	28	48.5 ± 10.9	2.8 (1.5–4.9)	18	5	5
	Prisma	27	47.0 ± 11.3	2.0 (1.5–5.0)	18	4	5
Basel	Prisma	69	42.0 ± 14.1	2.5 (1.5–4.0)	51	7	11
Total		124	45.0 ± 13.1	2.0 (1.5–4.5)	87	16	21

All patients underwent a single brain MRI acquisition at 3T (either MAGNETOM Skyra or MAGNETOM Prisma, Siemens Healthcare, Erlangen, Germany) in Lausanne; MRI in Basel were also acquired at 3T (MAGNETOM Prisma, Siemens Healthcare, Erlangen, Germany). In both centers, three-dimensional segmented echo-planar imaging (3D-EPI) (Sati et al., 2014), giving high-resolution T2*-*w* magnitude and phase images, and 3D T2-FLAIR images were acquired (Table 2). A 3D T1-weighted MPRAGE sequence was acquired in Lausanne and a 3D MP2RAGE in Basel (Marques et al., 2010). 3D-EPI images were obtained with a resolution of 0.65x0.65x0.65 mm^3^ and 0.67x0.67x0.67 mm^3^ in Lausanne and Basel hospitals, respectively. 3D FLAIR, MPRAGE, and MP2RAGE images were acquired with an isotropic resolution of 1 mm in both centers.

**Table 2:** Parameters of the MRI acquisition protocol followed in each center.

Hospital	Lausanne	Basel
Magnet strength	3T	3T
Manufacturer	Siemens	Siemens
Model	Prisma/Skyra	Prisma
Imaging plane	Sagittal	Sagittal

	3D-T2*-EPI	*3D-T2-FLAIR*	3D-T2*-EPI	*3D-T2-FLAIR*
Resolution (mm, isotropic)	0.65	1	0.67	1
N° of slices	288	176	256	176
Repetition time (TR, ms)	64	5000	64	5000
Echo time (TE, ms)	35	391	35	386
Inversion time (TI, ms)	–	1800	–	–
Flip angle (deg)	10	Variable	10	Variable
Averages	1	1	1	1
Acquisition time	6′ 20″	4′ 47″	6′ 19″	5′ 40″

### 2.2 Preprocessing steps

Three-dimensional MPRAGE or MP2RAGE were rigidly registered to the FLAIR space. Automated lesion segmentation was performed using a recently proposed deep learning architecture (La Rosa et al., 2020) which uses FLAIR and MP2RAGE to segment white matter and cortical lesions. In order to generate the lesion segmentation of those patients without the MP2RAGE available, the same network was re-trained with MPRAGE instead. For MPRAGE cases, segmentations were evaluated visually.

The post-processing and registration of unwrapped phase images were performed as previously described (Chavhan et al., 2009; Absinta et al., 2013). Three-dimensional (3D) FLAIR images, along with the lesion segmentations, were affinely registered to the T2* 3D-EPI space. Also, anatomical segmentations were generated with FreeSurfer (Fischl et al., 2002, Fujimoto et al., 2014) from MPRAGE or MP2RAGE images and affinely registered to the T2* 3D-EPI space. Lesion holes were filled with the lesion-filling function included in the FSL package (Battaglini, Jenkinson, and De Stefano 2012). All registrations were performed with the SimpleElastix tools (Marstal et al., 2016) by using the adaptive stochastic gradient descent optimizer together with the advanced Mattes mutual information metric (Mattes et al., 2001). The T2*-*w* 3D-EPI magnitude, the unwrapped 3D-EPI phase and the 3D FLAIR images will be hereafter referred as T2*, *phase*, and *FLAIR*, respectively.

### 2.3 Annotations of paramagnetic rim lesions

**Fig. 3:**
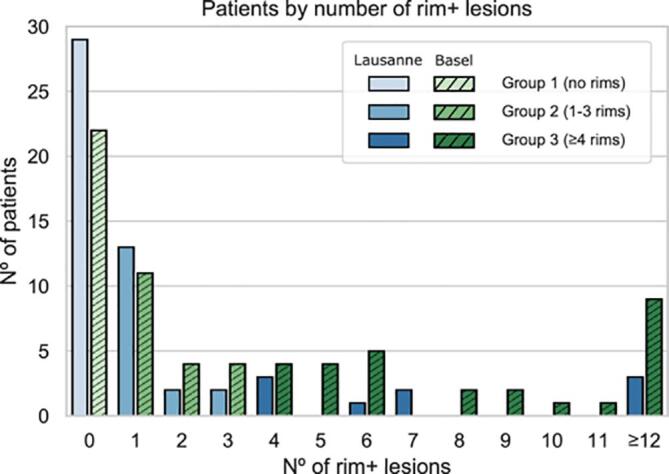
Distribution of patients according to their number of paramagnetic rim lesions (rim + lesions) in Lausanne and in Basel. The number (N°) of patients with 0, 1–3 and ≥ 4 paramagnetic rim lesions are reported for both Lausanne (scale of blue) and Basel (scale of green). (For interpretation of the references to colour in this figure legend, the reader is referred to the web version of this article.)

For training and evaluation of our deep-learning supervised classification method, a ground truth sample of rim+ and rim- was obtained (Yao et al., 2012). Rim+ lesions were manually annotated by two raters (PM and MA, with imaging research experience of 10 and 14 years, respectively), as summarized in Fig. 2A. For exploring the presence of rims, the phase images were primarily used, which helped to identify the phase shift mainly produced by iron-laden macrophages and relative myelin content at the lesion edge ( Absinta et al., 2016, Dal-Bianco et al., 2017). Moreover, 3D FLAIR was used in the annotation process to visually assess whether paramagnetic rims identified on the phase images matched an MS lesion on FLAIR, which allowed discarding potential false positives due to rim-shaped artefacts. After a first screening conducted individually by each expert, 38.3% of the lesions needed consensus review (Kappa score of 0.73 (Viera and Garrett 2005)), which was done in a second joint screening by the two experts. After consensus, 462 rim+ lesions were identified and further used in our study as the ground truth. The distribution of rim+ lesions per patient is shown in Fig. 3.

Rim- lesions were annotated as follows. Each connected component in the segmentation output (corresponding to the connected components by considering a 6-connected-voxels neighborhood) was considered a lesion candidate. All lesions that did not overlap with the rim + map were labeled as rim-. In order to have one lesion candidate paired with only one experts’ rim+ annotation, an experienced technician manually separated the rim+ lesions inside confluent ones.

A volume analysis performed with the lesions’ automatic segmentations revealed that all rim+ lesions included in our dataset were bigger than 12.3 mm^3^. As a result, we decided to exclude lesions smaller than 12.3 mm^3^ (1671 rim- lesions) from our study, as such small lesions could systematically be classified as rim- lesions. Interestingly, the volume analysis showed that rim+ lesions were, in general, bigger (325.2 ± 410.5 mm^3^) than rim- lesions (102.6 ± 231.8 mm^3^). However, these values must be cautiously interpreted as they were computed without manual corrections to better fit lesion borders, so real volumes could slightly differ.

Overall, our dataset of 124 patients contains 4857 rim- and 462 rim+ annotated lesions (10.5:1 ratio).

### 2.4 Patch extraction

Our motivation for a patch-based approach was based on two key factors. First, the experts’ decision relied exclusively on the appearance of the lesions and their close surroundings. Second, a patch-based approach allowed us to effectively deal with the class imbalance problem. As a counterpart, such an approach entails the extra challenge of choosing a suitable patch size. In our case, this patch needed to be big enough to cover most lesions while including only one lesion insofar as possible. We experimentally found out that a patch of size 28x28x28 voxels represented a good trade-off between both requirements. With a cube of such size, we managed to cover the 90% of the rim+ lesions at their full extent.

Hence, image patches were extracted centered on the center of mass of the automatically detected lesions and linearly normalized between −1 and 1 (Maggi et al., 2020). To ensure that the model was trained with reliable patches, we automatically removed lesions according to the following exclusion criteria. First, to make sure the whole rim was contained within the patch, lesions over 10,000 voxels were removed (32 rim- and 4 rim+ lesions). Lesions near air artefacts (discernible in phase) were also excluded (25 rim- and 1 rim+ lesions). Finally, to ensure that rim- patches did not also contain rim+ lesions, rim- patches with more than 900 voxels (410.5 mm^3^) belonging to rim+ lesions were removed (113 rim- lesions). The latter threshold was chosen considering the average volume of rim+ lesions. Thus, our training set included 4687 rim- and 457 rim+ lesions (10.3:1 ratio).

### 2.5 Network architecture

**Fig. 4:**
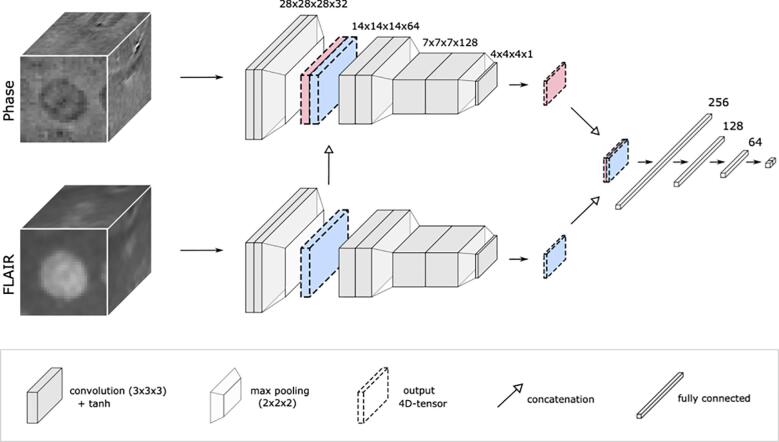
The architecture of RimNet. Built upon two parallel CNNs inspired by VGGNet, the proposed RimNet favors the multimodal low-level feature extraction by merging the output of the first convolutional block (two convolutions followed by a max pooling) of the second image to that of the main image (3D FLAIR and 3D-EPI phase in the figure, respectively). Finally, high-level multimodal feature maps are exploited through the final cascade of fully connected layers. Abbreviations: tanh, hyperbolic tangent function.

Our multimodal framework for the classification of rim+/rim- lesions is inspired by the expert’s imaging setting, which consists in the visual inspection of phase and FLAIR images. In this way, the prototype RimNet, see Fig. 4, is built upon two parallel CNNs based on the Visual Geometry Group Net (VGGNet) (Simonyan and Zisserman, 2015), which has proven to outperform other state-of-the-art architectures in similar multimodal approaches (Le et al., 2017). Each single CNN, or branch, receives a patch and feeds it to a succession of three blocks of two convolutional layers followed by a max-pooling layer. The main branch extracts phase features, which are merged with those extracted from FLAIR after the very first block in order to exploit multimodal low-level features. Finally, the two four-dimensional output tensors of each CNN are concatenated before being fed to a final succession of fully connected layers, which profits from the high-level multimodal features.

Phase features were needed as they better depict the presence/absence of paramagnetic rim. Additionally, we hypothesized that 1) FLAIR, with its optimal lesion-WM contrast, would provide lesion’s morphometric and location-aware features that could help in the rim+/- classification task; and 2) T2* could help in detecting rims with high paramagnetic effect. In order to validate these hypotheses, alternative RimNet configurations were also tested and compared: 1) replacing FLAIR by T2* (phase + T2*) and 2) using T2* instead of phase (T2* + FLAIR).

Along with the proposed multimodal scenarios, we also evaluated the prediction capability of each contrast separately and used these as baseline models. For this unimodal exploration, we used a network consisting of only one CNN branch of the RimNet directly connected to the cascade of fully connected layers.

### 2.6 Training strategy

Data augmentation is a well-known strategy for training deep neural networks to increase the performance in the testing phase. It is also a good strategy to tackle the class imbalance problem. Our preprocessed data augmentation consisted in rotating each rim+ lesion by 90°, 180°, and 270° in the three axes, which led to a tenfold increase of rim + and a 1.03:1 class ratio in the training set. Moreover, three elastically deformed versions of each lesion were generated, effectively quadrupling the training data. We also performed online data augmentation during the training first by flipping the patch along an axis (X, Y, Z, or none) and then by translating it 2 voxels (−2, 0 or +2) towards each of the three possible axes (X, Y, and Z). Both processes were designed to avoid generating repeated patches and hence increase the generalization of our model.

In order to avoid overfitting and to better reflect the performance in a clinical scenario, models were trained following a per-site stratified four-fold nested cross-validation procedure. The stratification process took into consideration the number of samples per class (rim+/-) and per center included in each fold. To perform a patient-wise rim analysis simulating a real case scenario, we imposed that all lesions of the same patient had to belong to the same split. This yielded folds each with 36.3 ± 1.9 and 78.0 ± 0.0 rim+ lesions (492.8 ± 4.6 and 679.0 ± 4.9 rim- lesions) from Lausanne and Basel, respectively. Regarding the number of patients, each fold contained 13.8 ± 0.8 from Lausanne and 17.2 ± 3.0 from Basel. All experiments were trained with this fold configuration.

In RimNet, each branch was trained with its own weights. The training of our models was conducted as follows: the initial weights were drawn from Xavier initialization (Glorot and Bengio, 2010), a hyperbolic tangent (*tanh*) was used as activation function, batch normalization was applied, and loss minimization was performed by the ADAM optimizer (Kingma and Ba, 2017), along with learning rate decay and early stopping. For each fold’s distribution, a three-fold inner cross-validation determined the number of epochs trained with each learning rate ($1.0\cdot 10^{-4},5.0\cdot 10^{-5},2.5\cdot 10^{-5},1.0\cdot 10^{-5}$) before its decay, which was triggered after three consecutive epochs without a decrease in the validation loss. Early stopping was applied when the last learning rate change was triggered. For all network configurations, training was done with a batch size of 32 and SoftMax cross-entropy as the loss function.

In order to evaluate the generalization of RimNet across different clinical centers, we additionally performed an inter-scanner/hospital study where the network was trained with only the Basel patients and then tested on the Lausanne dataset. The cross-validation process yielded four models trained with Basel data, which were used as an ensemble of classifiers to infer the labels for Lausanne patients’ lesions.

### 2.7 Statistical evaluation

Receiver operating and precision-recall curves (ROC and PR, respectively) of the different testing folds were interpolated (piecewise constant interpolation) and averaged to show the overall performance at the lesion-level of the trained network configurations. For each curve, the area under the curve value (AUC) was computed by averaging the four AUC values across the testing folds. A comparison of ROC curves among different network configurations was carried out using the DeLong test (DeLong, DeLong, and Clarke-Pearson, 1988), using the implementation included in the pROC package in R (“R-Project, Version 3.6.2.” n.d.) (Robin et al., 2011).

A patient-level analysis was also performed. The performance of the experts was evaluated by comparing their individual pre-consensus annotations with the ground truth. To do so, we set the operating point so it yielded a specificity of 95%, and we categorized patients as “chronic active” and “non-chronic active” based on the total number of rim + lesions per patients, following a previous study that observed higher disability in patients with four lesions or more than those with fewer ( Absinta et al., 2019, Maggi, 2020).

In both lesion- and patient-wise analysis, sensitivity, specificity, positive predictive value (PPV), and negative predictive value (PPV) were calculated from the confusion matrices and compared using the McNemar test with continuity correction. P-value < 0.05 was considered statistically significant.

### 2.8 Error analysis

The lack of established international consensus criteria on the rim+/- classification problem leads to low inter-rater reliability. A disagreement between both raters prior to consensus could be interpreted as a low-confidence indicator of their joint decision. To understand the challenges that these ambiguous lesions posed to our method, we divided our lesions into two subsets based on the existence or not of an initial agreement on both raters’ decision and compared the performance of RimNet (phase + FLAIR) on both. The comparison was done by using the independent samples T-test without assuming equal variance.

Additionally, a second rating was carried out for the lesions misclassified by RimNet with a certainty value over 95%, according to the probability inferred by the network’s last layer. To do so, experts worked blinded to each other but knew both the current ground truth label and the prediction of RimNet. After this independent re-evaluation, experts reached consensus during a joint session for the lesions where they had initially disagreed. RimNet errors were classified into: 1) true network mistakes, 2) ground truth mistakes (lesions missed by the experts or where experts changed their rating based on RimNet’s decision) and 3) incorrect candidate lesion selection, specifically rim- lesions that, according to experts, should not be considered as such due to confluence with rim+ lesions.

Finally, anatomical segmentations were used to analyze the performance of RimNet depending on lesion location. Five patients were excluded from this analysis because of segmentation or registration issues. The regions-of-interest included were cerebrum deep white matter, cortex, ventricles, deep gray matter, brainstem and cerebellum. The cortex and the ventricles were dilated by 2 mm and 3 mm, respectively, according to previous definitions of periventricular and juxtacortical MS lesions (Jehna et al., 2015, Filippi et al., 2019). An overlap of at least half of the lesion’s volume with a region of interest was required to categorize the lesion as belonging to that respective region except for the periventricular white matter, where any overlap was considered. The classification performance of RimNet was evaluated in each region.

## 3 Results

### 3.1 Lesion-wise analysis

**Fig. 5:**
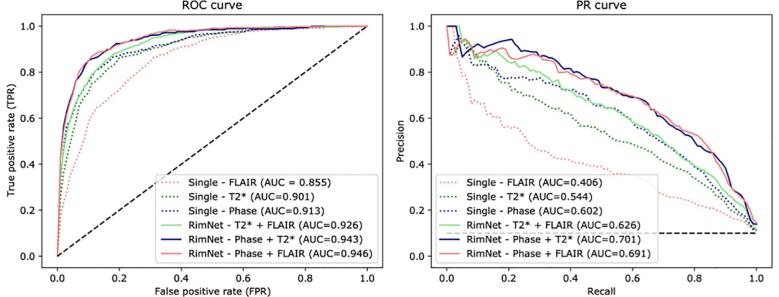
ROC and PR curves for all network configurations. Abbreviations: ROC, receiver operating characteristic; PR, precision-recall; ICS, inter-center study; Single, unimodal network; RimNet, the proposed multimodal network.

Results of the lesion-wise analysis for all single and multimodal tested architectures are shown in Fig. 5. The prior exploration of the prediction capabilities of each individual modality shows that all modalities can, with different contributions, predict rim + lesions substantially better than chance. As expected, both susceptibility-based modalities performed better than FLAIR (AUC = 0.855) at classifying rim+ lesions (P's < 0.0001). Thus, phase (AUC = 0.913) and T2* (AUC = 0.901) position themselves as the best sequences for this task (P = 0.47, DeLong test).

**Fig. 6:**
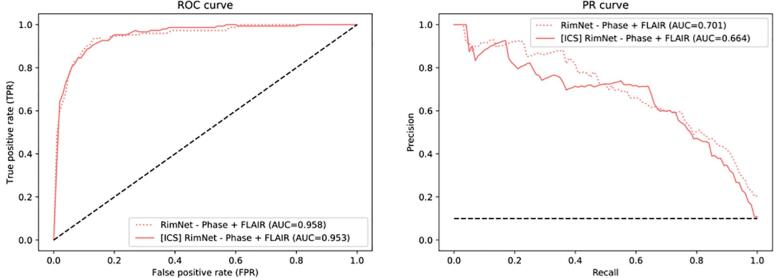
ROC and PR curves of RimNet in the inter-center study. Results are compared to those of RimNet trained with data from both centers and evaluated in Lausanne (P = 0.47). Abbreviations: ROC, receiver operating characteristic; PR, precision-recall; ICS, inter-center study; RimNet, the proposed multimodal network.

The prototype RimNet was evaluated with three different combinations of modalities as input: phase + FLAIR (AUC = 0.946), phase + T2* (AUC = 0.943), and T2* + FLAIR (AUC = 0.926). All combinations clearly outperformed the best unimodal architecture (P values of < 0.0001, <0.0001, and 0.0183, respectively). At the same time, bimodal combinations of phase and either T2* or FLAIR showed significantly higher prediction capabilities than the T2* and FLAIR combination (P values of 0.003 and 0.023, respectively). No statistically significant differences were found between phase + T2* and phase + FLAIR (P = 0.48), so hereafter we will call the proposed network with the latter inputs’ configuration RimNet. In the inter-center study evaluation (Fig. 6), RimNet trained only with Basel samples showed a performance (AUC = 0.953) indistinguishable from the same network configuration trained with samples from both centers (AUC = 0.958, P = 0.47).

The lesion-wise performance metrics of the best single architecture (phase) and RimNet were computed on the specified operating point and compared to experts in Table 3. Results for the inter-center study are also included. For the cross-validation configuration, the single modality model performed with a sensitivity of 62.1%, a specificity of 95.0%, a PPV of 53.9%, and an NPV of 96.3%, while RimNet performed with a sensitivity of 70.6%, a specificity of 94.9%, a PPV of 56.9%, and an NPV of 97.1%. In the inter-center study, RimNet performed with a sensitivity of 75.8%, a specificity of 95.1%, a PPV of 52.8%, and an NPV of 98.3%. In both scenarios, lesion-wise performance for both raters was superior to the one of the evaluated models (McNemar’s test; P’s < 0.0001).

**Table 3:** Lesion-wise results of best single and bimodal architectures, compared to both experts, for the cross-validation and the inter-center scenarios. Abbreviations: PPV, positive predictive value; NPV, negative predictive value; P’s (#1), p-values relative to expert #1; P’s (#2), p-values relative to expert #2.

Lesion-wise results	Accuracy	F1	Sensitivity	Specificity	PPV	NPV	P’s (#1)	P’s (#2)
Cross-validation evaluation								
Single phase model	91.3	57.6	62.1	95.0	53.9	96.3	<0.0001	<0.0001
RimNet: phase + FLAIR	94.6	63.0	70.6	94.9	56.9	97.1	<0.0001	<0.0001
Expert #1	97.9	86.4	77.9	99.8	97.0	97.9		1.000
Expert #2	97.8	85.6	77.5	99.7	96.5	97.9	1.000	
Inter-center study								
RimNet: phase + FLAIR	93.8	62.3	75.8	95.1	52.8	98.3	<0.0001	<0.0001
Expert #1	98.7	89.6	83.9	99.8	96.2	98.8		0.61
Expert #2	98.1	84.7	77.9	99.6	92.8	98.4	0.61	

### 3.2 Patient-wise analysis

**Fig. 7:**
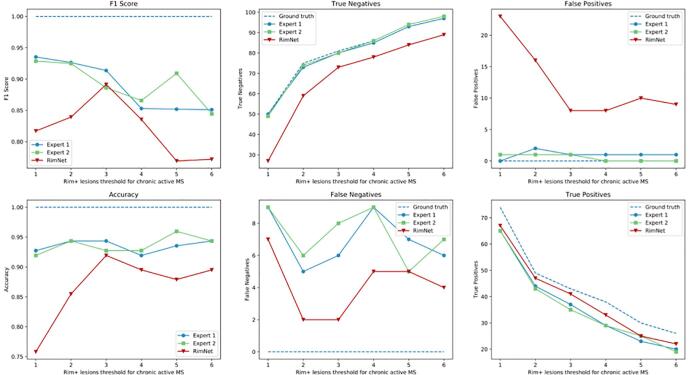
Patient-wise analysis depending on the number of paramagnetic rim lesions set to consider a patient as “chronic active.” RimNet (with phase and FLAIR as inputs) is evaluated choosing an FPR of 0.05. In the first column, comparison with individual expert performance. In the middle and right columns, absolute values for missed and correct predictions showing the comparison between RimNet and the experts’ assessment.

**Fig. 8:**
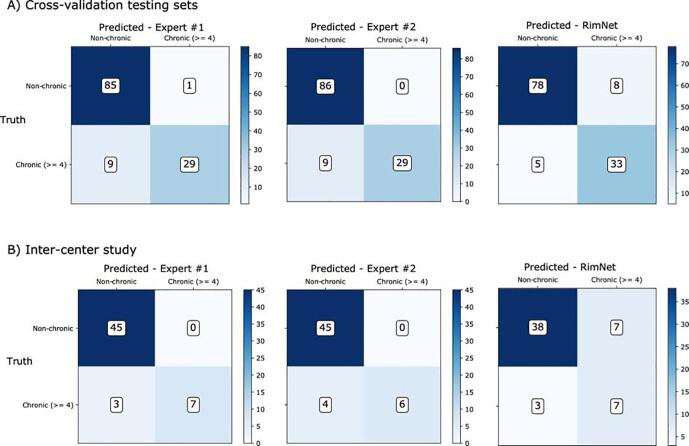
Confusion matrices of RimNet (phase + FLAIR) compared to those of the experts. A) shows the results of RimNet trained with cross-validation using all the data. B) shows the results of the inter-center study, in which the model is trained with Basel data and evaluated as an ensemble of classifiers with patients from Lausanne.

We evaluated the RimNet patient classification performance based on the number of rim + lesions per patient. To do so, we kept the operating point at a lesion-wise accepted false positive rate (FPR) of 0.05 and we categorized patients as “chronic active” and “non-chronic active” based on the total number of rim+lesions per patient by using thresholds ranging from 1 to 6. Each patient’s rim analysis took an average of 300 ms on a desktop Intel(R) Core i7-4790 CPU machine at 3.60 GHz and a GeForce GTX 1080Ti GPU. The results are shown in Fig. 7. The exact values for accuracy and F1 scores can be found in the supplementary materials, Table 1. Significant differences between the model and the raters were proved only for the 1-rim-lesion threshold when compared to expert #1 (for the 1 to 6 thresholds, p-values of 0.0034, 0.0604, 0.2284, 0.1770, 0.1524, 0.1839) and for the 1- and 2-rim-lesions thresholds when compared to expert #2 (p-values of 0.0056, 0.0344, 0.14207, 0.1480, 0.2116, 0.0961). Based on our patient-level performance analysis and on recent evidences from the literature ( Absinta et al., 2019, Maggi, 2020), we computed the confusion matrices using the 4-rim + lesions threshold (≥4 paramagnetic rim lesions per patient), for both the cross-validation and the inter-center scenario (Fig. 8). According to this threshold, 35.4% and 22.2% of patients from Basel and Lausanne, respectively, presented chronic active MS.

### 3.3 Error analysis

**Fig. 9:**
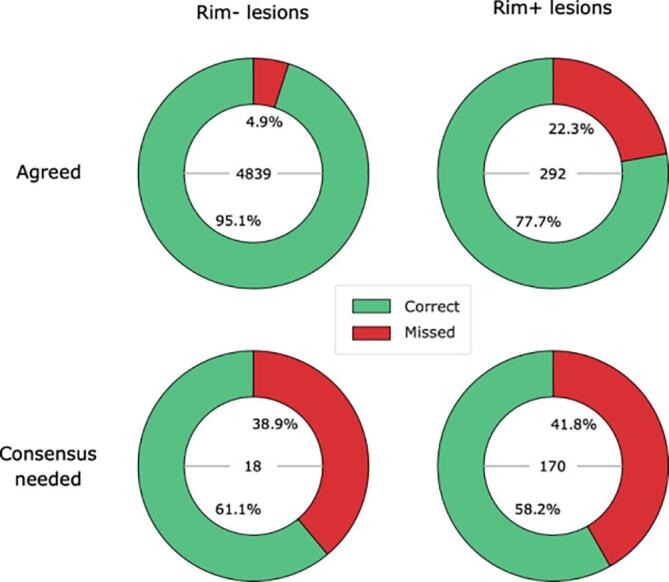
RimNet errors analysis. The proportion of RimNet correct rim+/- predictions based on whether a consensus was required after the individual experts’ annotations (consensus needed, bottom row) or not (agreed, top row) is shown. The columns split lesions based on their ground truth label (rim+ and rim- for the presence or absence of a paramagnetic rim, respectively). The total number of lesions of each type is shown inside the pie charts.

After the first individual screening of the annotation process, 188 lesions needed consensus review. During the joint session, experts agreed to classify 170 as rim+ (90.4%) and 18 as rim- (9.6%). Fig. 9 shows the ratios of RimNet’s mistakes split by whether the lesions required consensus in the annotation process or not. Results show how RimNet (phase + FLAIR) misclassified significantly (p < 0.0001) more rim+ lesions that required consensus (41.8%) than rim + lesions that did not (22.3%). The same behavior was observed with rim- lesions, for which RimNet misclassified 38.9% of rim- lesions requiring consensus, compared to a miss rate of 4.9% for those lesions with an early agreement (p = 0.01).

Experts re-rated 47 false positive (FP) and 36 false negative (FN) lesions corresponding to rim- and rim+ labeled lesions, respectively. Inter-rater reliability was measured with the kappa coefficient, which was 0.36 and 0.68 for FN and FP lesions, respectively. Twenty-two FP (46.8%) were considered mistakes of the automatic rim- lesion selection and two FN (5.5%) lesions had segmentation issues. Based on the RimNet assessment, experts changed the ground truth decision for 14 rim- lesions (29.8% of FP cases) and 4 rim+ lesions (11.1% of FN cases). Therefore, experts confirmed their initial decision for 11 FP (23.4%) and 29 FN (80.5%) lesions.

**Fig. 10:**
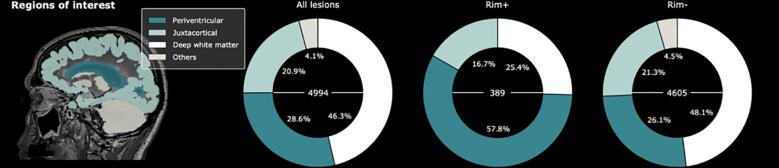
Anatomical distribution of lesions based on the presence or absence of the paramagnetic rim (rim+ and rim-, respectively). Four regions of interest where considered in this analysis: periventricular, juxtacortical, deep white matter and others (cerebellum, brain stem and deep gray matter). The total number of lesions of each type is shown inside the pie charts.

Three hundred and eighty-nine rim+ and 4605 rim- lesions were classified in terms of their anatomical location within the brain. Most rim+ lesions were located in the periventricular white matter (p < 0.0001, computed with the one-tailed two-proportion z-test). Results in Fig. 10 show: 65 (16.7%) rim+ and 981 (21.3%) rim- lesions were near the cortex (juxtacortical), 225 (57.8%) rim+ and 1203 (26.1%) rim- lesions were within the periventricular white matter, and 99 (25.4%) rim+ and 2215 (48.1%) rim- lesions were in the rest of white matter. The deep gray matter, the cerebellum and the brain stem regions only included a total of 43 (0.9%), 60 (1.3%), and 103 (2.2%) rim- lesions, respectively, and none of them included rim+ lesions. The error analysis yielded accuracy and F1 values of 96.3% and 61.2% for deep white matter lesions, 92.8% and 53.4% for juxtacortical lesions and 88.7% and 67.2% for periventricular lesions, respectively. Positive predictive values reported were 55.8%, 44.8%, and 61.7% for deep white matter, juxtacortical and periventricular lesions, respectively. RimNet only missed one rim- lesion outside these three areas.

## 4 Discussion

Here we propose “RimNet,” a deep-learning prototype for automatic assessment of paramagnetic rim lesions in MS. By exploiting different MRI contrasts in a multi-center and multi-scanner setting, we showed that RimNet performance is at the level of expert readers. Importantly, the proposed RimNet prototype achieves remarkably good paramagnetic rim lesion detection results even when tested in a multi-center scenario, thus supporting its potential for generalization across different clinical centers and datasets.

Among the different MRI techniques so far proposed to detect paramagnetic rim lesions in MS (Haacke et al., 2009, Yao et al., 2012, Walsh et al., 2013, Absinta et al., 2018, Clarke et al., 2020), 3D-EPI derived unwrapped phase images adopted in this study have shown promising performance in depicting the phase shifts produced by iron-laden macrophages and relative myelin content at the lesion edge and have been implemented for visual rim+/- analysis in clinical MRI studies (Absinta et al., 2018; Absinta et al., 2019). Our automated rim evaluation is in line with these findings, as illustrated in our experiments on the single modality network, which showed the best performance when using phase as input modality (AUC = 0.913). Although containing susceptibility and morphological information of MS lesions, T2*-magnitude did not prove as reliable as phase in the manual rim + classification (Bian et al., 2013, Absinta et al., 2018). However, the T2* network (AUC = 0.901) showed a surprisingly good performance, closer than expected to the phase one (p = 0.470). Nowadays, the role of FLAIR images during visual rim assessment is mainly restricted to the detection of MS lesions, thus allowing the experts to discard potential false positives due to rim-shaped artefacts. Although one could expect poor performance using only FLAIR as input, our experiments show, on the contrary, that FLAIR’s prediction capabilities are far from insignificant (AUC = 0.855). This relatively good performance of unimodal FLAIR architecture, along with the notably good performance of T2*, suggests that, beyond the presence or absence of a lesion, morphometric features such as size, shape, and signal intensity, as depicted by these modalities, could play an important role in the classification of rim+/- lesions.

As an improvement on the simple unimodal approaches, we present the prototype RimNet, which relies on 3D multimodal MRI input patches. The early fusion of low-level features extracted from FLAIR and phase allows RimNet to extract low-level multimodal and lesion-aware features from the latter. Simultaneously and thanks to the straightforward parallel flow of the data of both modalities, the network benefits from the prediction capacities of both contrasts. High-level multimodal capabilities are exploited through the last fully connected layers. Results showed the superior performance of RimNet over all unimodal architectures (AUC = 0.946). When replacing phase with T2*, performance dropped significantly (AUC = 0.926), thus validating the hypothesis that T2* is not as reliable as phase regarding the extraction of rim + features. Also, the almost negligible loss of performance when replacing FLAIR with T2* (AUC = 0.943) supports the hypothesis that both FLAIR and T2* can be equally used to extract morphometric features that enhance the overall classification performance. This conclusion suggests the feasibility of performing rim+/- analysis with only one single MRI acquisition, although we chose to use the conventional FLAIR images in most of the analyses in the current work.

In the lesion-wise analysis, RimNet showed excellent performance with regard to sensitivity (70.6%) and negative predictive value (NPV, 96.3%), which are values close to those of the experts (77.7% and 97.9%, averaged values for experts’ sensitivity and NPV values, respectively). The downside of RimNet is its low positive predictive value (56.9%, compared to 96.5% and 97% of the experts), which is due to a relatively high number of false positives. However, this does not prevent it from becoming an excellent assessment tool to assist the visual rim analysis, as in this scenario lesions classified as rim+ would always be checked by an expert. The biggest obstacle that MRI deep learning techniques face in their way toward being included in clinical workflows is the need for robustness against changes in the acquisition hardware and software. In other words, they must prove a good capability of generalizing with data acquired in different settings across healthcare institutions. The outstanding performance of RimNet in the inter-center study (AUC = 0.953) places it as a promising decision support tool for physicians in the rim analysis of MS patients, by providing an accurate rim+ estimation in less than a second. This could be integrated with the only requirement of an expert clicking on the lesion in order to automatically extract and analyze the patch, instantly obtaining the decision of an extra rater. This would help to reduce the effect of the interobserver variability and increase the overall accuracy in rim analysis.

The potential of RimNet so conceived is reinforced by the results of the RimNet-assessed rating, which was performed in a subset of high-confidence RimNet mispredictions. Excluding the lesions that correspond to lesion selection mistakes and therefore would not affect the extra-rater scenario, experts changed their initial decision and agreed on the existence of a paramagnetic rim for 56% of the re-rated rim- lesions. The analysis regarding the anatomical location of the lesions can give us a slight intuition on the origin of these mistakes. In the first place, the low positive predictive value of RimNet on juxtacortical lesions (44.8%), compared to WM (55.8%) or periventricular (67.2%) lesions suggests that cortical folds and susceptibility artefacts could represent an important source of false positives. Also, the relatively low accuracy shown for periventricular lesions (88.7%), mainly due to a high number of false positives (8.6%), could have its origin in the lesion selection mistakes identified during the RimNet-assessed rating.

This conception of RimNet is also supported by its results in the patient-wise analysis. Considering the recently proposed, clinically meaningful threshold of ≥ 4 rim + lesions per patient (Absinta et al., 2019; Maggi, 2020), RimNet achieves a higher sensitivity (83.5%) and negative predictive value (94.0%) than experts (76.3% and 90.5%, respectively, averaged across experts), as well as similar accuracy (89.5%) and F1 score (83.5%) (92.3% and 85.8%, respectively, averaged across experts). As already depicted from the lesion-wise analysis, the main weaknesses of RimNet are its sensitivity and positive predictive values. Nonetheless, the absence of a drop in performance in the patient-wise inter-center study further supports the potential of RimNet as decision-support tool. However, the patient-wise results need to be interpreted with caution given the relatively small size of our cohorts and the differences in the proportion of patients with ≥ 4 rim + lesions across centers (35.4% and 22.2% for Basel and Lausanne, respectively).

The largest limitation of our method resides in the very nature of any patch-based approach: lesions need to fit in patches of a fixed size. As a result, big lesions and confluent lesions entail big challenges. In the presented approach, the former were fed to the network untouched and the latter were manually split into unique lesions. This represents an obstacle to full automation of the rim analysis, which is highly needed for the inclusion of RimNet in clinical practice. Future work should improve our pipeline, so it becomes a fully automated approach. Another important limitation of our work resides in the lack of notable differences among the acquisition protocols of the scanners included in the inter-center study. Thus, although the inter-center study can be considered a preliminary analysis of the RimNet generalization power, future studies should test the performance of RimNet using data acquired with different gradient-echo MRI sequences. Finally, we only focused on FLAIR and T2*-EPI sequences. Future work should explore other MRI modalities such as T1-weighted or quantitative susceptibility maps (QSM) (Wisnieff et al., 2015, Kaunzner et al., 2019), which could provide RimNet with new information on the tissue properties of paramagnetic rim lesions.

In conclusion, RimNet is the first deep learning-based framework to automatically classify MS lesions based on the presence/absence of a paramagnetic rim. Its excellent performance holds great promise for the translation of the paramagnetic rim lesion biomarker in everyday clinical practice.

## 5 Conflicts of interest and source of funding

Francesco La Rosa is supported by the European Union's Horizon 2020 research and innovation program under the Marie Sklodowska-Curie project TRABIT (agreement No 765148).

Hamza Kebiri is supported by the Swiss National Science Foundation (SNSF project 205321-182602).

Po-Jui Lu is supported by the Swiss National Science Foundation (SNSF grant grantPP00P3_176984).

Dr. Matthias Weigel is paid by the Swiss National Science Foundation (SNSF grant PP00P3_176984).

Dr. Mário João Fartaria is full-time employee of Siemens Healthcare AG and owns shares in Siemens Healthcare AG.

Dr. Tobias Kober is full-time employee of Siemens Healthcare AG and owns shares in Siemens Healthcare AG.

Dr. Pascal Sati, Dr. Daniel S. Reich, and Dr. Martina Absinta are supported by the Intramural Research Program of the National Institute of Neurological Disorders and Stroke, National Institutes of Health, Bethesda, Maryland, USA.

Dr. Martina Absinta is supported by the Conrad N. Hilton Foundation (grant# 17313).

Dr. Cristina Granziera is funded by the Swiss National Science Foundation (SNSF) grant PP00P3_176984, the Stiftung zur Förderung der gastroenterologischen und allgemeinen klinischen Forschung, EUROSTAR E!113682 HORIZON2020.

For the remaining authors none were declared.

## CRediT authorship contribution statement

**Germán Barquero:** Conceptualization, Methodology, Software, Validation, Formal analysis, Investigation, Data curation, Writing - original draft, Visualization. **Francesco La Rosa:** Conceptualization, Methodology, Software, Validation, Formal analysis, Investigation, Data curation, Writing - original draft. **Hamza Kebiri:** Conceptualization, Methodology, Software, Validation, Formal analysis, Investigation, Data curation, Writing - original draft. **Po-Jui Lu:** Resources, Data curation, Writing - review & editing. **Reza Rahmanzadeh:** Resources, Writing - review & editing. **Matthias Weigel:** Resources, Writing - review & editing. **Mário João Fartaria:** Investigation, Writing - review & editing. **Tobias Kober:** Investigation, Writing - review & editing. **Marie Théaudin:** Resources, Writing - review & editing. **Renaud Du Pasquier:** Resources, Writing - review & editing. **Pascal Sati:** Validation, Formal analysis, Investigation, Writing - review & editing. **Daniel S. Reich:** Validation, Formal analysis, Investigation, Writing - review & editing. **Martina Absinta:** Validation, Formal analysis, Investigation, Resources, Data curation, Writing - review & editing. **Cristina Granziera:** Conceptualization, Methodology, Validation, Investigation, Resources, Writing - original draft, Writing - review & editing, Supervision, Project administration, Funding acquisition. **Pietro Maggi:** Conceptualization, Methodology, Validation, Investigation, Resources, Data curation, Writing - original draft, Writing - review & editing, Supervision, Project administration. **Meritxell Bach Cuadra:** Conceptualization, Methodology, Validation, Investigation, Resources, Writing - original draft, Writing - review & editing, Supervision, Project administration, Funding acquisition.
