# Supplementary material for: RimNet: A deep 3D multimodal MRI architecture for paramagnetic rim lesion assessment in multiple sclerosis
Source: Neuroimage Clin. 2020 Sep 4;28:102412. doi: 10.1016/j.nicl.2020.102412 (PMC7509077; doi:10.1016/j.nicl.2020.102412)
Supplement: Supplementary Data 1 [file mmc1.docx]

| Accuracy | ≥ 1 | ≥ 2 | ≥ 3 | ≥ 4 | ≥ 5 | ≥ 6 |
| --- | --- | --- | --- | --- | --- | --- |
| RimNet: phase + FLAIR | 75.8 | 85.5 | 91.9 | 89.5 | 87.9 | 89.5 |
| Expert #1 | 92.7 | 94.4 | 94.4 | 91.9 | 93.5 | 94.4 |
| Expert #2 | 91.9 | 94.4 | 92.7 | 92.7 | 96.0 | 94.4 |
| F1 score | | | | | | |
| RimNet: phase + FLAIR | 81.7 | 83.9 | 89.1 | 83.5 | 76.9 | 77.2 |
| Expert #1 | 93.5 | 92.6 | 91.4 | 85.3 | 85.2 | 85.1 |
| Expert #2 | 92.9 | 92.5 | 88.6 | 86.6 | 90.9 | 84.4 |

(2 columns) **Table 1.** **Comparison of the patient-wise accuracy and F1 score values for RimNet and experts.** Columns correspond to the number of paramagnetic rim lesions set to consider a patient as “chronic active.”
